# HIIT Models in Addition to Training Load and Heart Rate Variability Are Related With Physiological and Performance Adaptations After 10-Weeks of Training in Young Futsal Players

**DOI:** 10.3389/fpsyg.2021.636153

**Published:** 2021-01-22

**Authors:** Fernando de Souza Campos, Fernando Klitzke Borszcz, Lucinar Jupir Forner Flores, Lilian Keila Barazetti, Anderson Santiago Teixeira, Renan Felipe Hartmann Nunes, Luiz Guilherme Antonacci Guglielmo

**Affiliations:** ^1^Physical Effort Laboratory, Federal University of Santa Catarina, Florianópolis, Brazil; ^2^Department of Physical Education, State University of Western Parana, Marechal Cândido Rondon, Brazil

**Keywords:** sports, physical performance, high intensity interval training, shuttle-run, sprint

## Abstract

**Introduction:**

The present study aimed to investigate the effects of two high-intensity interval training (HIIT) shuttle-run-based models, over 10 weeks on aerobic, anaerobic, and neuromuscular parameters, and the association of the training load and heart rate variability (HRV) with the change in the measures in young futsal players.

**Methods:**

Eleven young male futsal players (age: 18.5 ± 1.1 years; body mass: 70.5 ± 5.7 kg) participated in this study. This pre-post study design was performed during a typical 10 weeks training period. HIIT sessions were conducted at 86% (HIIT_86_; *n* = 6) and 100% (HIIT_100_; *n* = 5) of peak speed of the FIET. Additionally, friendly and official matches, technical-tactical and strength-power training sessions were performed. Before and after the training period, all players performed the FIET, treadmill incremental, repeated sprint ability (RSA), sprint 15-m, and vertical jump tests (CMJ and SJ), and the HRV was measured. Training load (TL) was monitored using the session rating of perceived effort. Data analysis was carried out using Bayesian inference methods.

**Results:**

The HIIT_86_ model showed clear improvements for the peak oxygen uptake (VO_2_peak), peak speed in the treadmill incremental test, first and second ventilatory thresholds, RSA best and mean times, CMJ, and SJ. The HIIT_100_ model presented distinct advances in VO_2_peak, peak speed in the treadmill incremental test, RSA mean time, and CMJ. Between HIIT models comparisons showed more favorable probabilities of improvement for HIIT_86_ than HIIT_100_ model in all parameters. TL data and HIIT models strongly explained the changes in the RSA mean and best times (*R*^2^ = 0.71 and 0.87, respectively), as well as HRV changes, and HIIT models explained positively VO_2_peak changes (*R*^2^ = 0.72). All other changes in the parameters were low to moderately explained.

**Conclusion:**

The HIIT_86_ proved to be more effective for improving aerobic, RSA, and neuromuscular parameters than HIIT_100_ during a typical 10-week futsal training period. So, strength and conditioning specialists prescribing shuttle-run intermittent exercises at submaximal intensities can manage the individual acceleration load imposed on athlete increasing or decreasing either the set duration or the frequency of change of direction during HIIT programming.

## Introduction

Futsal is a team sport involving a complex range of high-intensity locomotor activities, requiring both aerobic and anaerobic fitness to cope with the multiple requirements of the match (Ribeiro et al., 2020). Research studies investigating this sport modality have increased significantly over the past two decades (Barbero-Alvarez et al., 2008; Castagna et al., 2009; De Oliveira Bueno et al., 2014; Caetano et al., 2015; Nakamura et al., 2020; Ribeiro et al., 2020), contributing to better understanding of the physical and skills requirements during the futsal match and organization of the training contents. Time motion analysis studies reported that during a single match, futsal players cover, on average, a total distance of 3000–4000 m, of which 8–13 and 5–9% are performed at high-speed running (HSR; >15.5 km/h) and sprinting (>18.3 km/h), respectively (Barbero-Alvarez et al., 2008; Castagna et al., 2009; De Oliveira Bueno et al., 2014). Repeated sprint sequences (RSS) with up to 2 to 3 sprints interspersed with 15 s of recovery are also frequently (∼80 occurrences per match) performed in futsal (Caetano et al., 2015). Similarly, the execution of deceleration and acceleration actions constitutes a critical part of the futsal players’ work-rate (Ribeiro et al., 2020). Thus, HSR episodes, sprints, RSS, changes of directions (COD), and acceleration and deceleration actions are among the main types of activities that players should be prepared to perform efficiently. This information may be of practical relevance for the design of suitable training programs in order to enhance the main physical capacities related to successful performance in futsal.

The training intensity is among the first training variables to be manipulated in most physical conditioning programs for athletes (Buchheit and Laursen, 2013). The definition of number and duration of sets performed per training session is also a key component to determine the total training volume (i.e., total work duration performed) (Buchheit and Laursen, 2013). Thus, any manipulation in these variables, in an isolated or combined manner, will be decisive to determine the magnitude of adaptations to training (Buchheit and Laursen, 2013). High-intensity interval training (HIIT) and repeated-sprint/sprint interval training (RST/SIT) are currently among the most frequently used training models in physical conditioning programs of team sports players (Buchheit et al., 2008; Ferrari-Bravo et al., 2008; Buchheit and Laursen, 2013; McGinley and Bishop, 2016). The current evidence comparing RST/SIT vs. HIIT models demonstrates conflicting results, with some studies showing reduced gains (Buchheit et al., 2008) and others superior gains (Ferrari-Bravo et al., 2008) in performance after RST/SIT compared to HIIT models. Other studies have examined the effects of two work-matched HIIT models performed at different training intensities (McGinley and Bishop, 2016; Viaño-Santasmarinas et al., 2018). For instance, Viaño-Santasmarinas et al. (2018) did not find any improvements in aerobic fitness indices and RSA performance after two work-matched, HIIT models, performed at 85 and 95% of the final speed obtained in the 30–15 Intermittent Fitness Test (V_IFT_) in professional handball players. This finding is in line with other research reporting no effect of training intensity on RSA performance outcomes following HIIT models (McGinley and Bishop, 2016). Thus, these two studies concluded that distinct training intensities during HIIT models produce similar performance enhancements when the same total exercise duration (i.e., *isotime*) is applied. While this *isotime* approach is a methodological strategy used in several studies to investigate the isolated effect of training intensity (McGinley and Bishop, 2016; Teixeira et al., 2018, 2019; Viaño-Santasmarinas et al., 2018), few studies have addressed the potential effects of distinct HIIT models varying in exercise intensity and total work duration on the athletic performance of team sport players (Ferrari-Bravo et al., 2008; Maggioni et al., 2019). Prior research studies investigating the integrative effects of these two key variables on the adaptive responses to distinct HIIT formats are limited to individual endurance sports athletes (Seiler et al., 2013).

Considering the multidirectional running pattern during futsal matches, HIIT strategies are usually composed of shuttle-runs in order to increase the specificity of these HIIT drills (Akenhead et al., 2014; Teixeira et al., 2018, 2019). In this sense, the simultaneous manipulation of the exercise intensity and total work duration during shuttle run HIIT models will have a direct influence on the magnitude and total number of acceleration and deceleration actions performed for each COD during a training session, respectively (Buchheit and Laursen, 2013; Akenhead et al., 2014). For instance, shuttle-run drills with a greater frequency of COD elicit an increased metabolic, cardiovascular, neuromuscular, and perceptual response in team sport athletes (Akenhead et al., 2014). Of interest, the number of COD performed during a short-term training period (5–6 weeks) was shown to be crucial to improve a variety of physical performance parameters in female team sport players (Sanchez-Sanchez et al., 2018; Teixeira et al., 2019). This research topic still needs further investigation, since the same effects observed in female athletes have not been noticed in male athletes (da Silva et al., 2015; Attene et al., 2016). Therefore, comparative studies examining the effects of two HIIT models at submaximal and maximal intensities with a contrasting total work duration in male futsal players could address practical questions on whether shuttle run HIIT programs, taking into account the physiological consequences of COD (Akenhead et al., 2014; Teixeira et al., 2018), would be more effective if performed at lower intensities with longer sets or at higher intensities with shorter set durations.

The dose-response relationship between the accumulated training load (TL) and performance adaptations is another relevant topic in the field of team sports, which deserves attention from coaches and sport scientists. The majority of studies examining the dose-response relationship have been primarily conducted with soccer and rugby players (Taylor et al., 2018; Daniels et al., 2019; Rabbani et al., 2019; Ellis et al., 2020). To date, few studies have investigated the dose-response relationship between TL and performance adaptations in futsal (Oliveira et al., 2013; Nakamura et al., 2020). Oliveira et al. (2013) did not find any association between TL and heart rate variability (HRV) and aerobic-anaerobic performance changes during seasonal training phases in professional futsal players. Differently, Nakamura et al. (2020) showed that accumulated TL negatively affected the physical and physiological adaptations of elite futsal players. Thus, further studies are still warranted to better understand the potential consequences of accumulated TL during the preseason phase on the subsequent changes in performance in futsal teams, especially in youth players.

The current study aimed to compare the effects of two shuttle run HIIT models performed at 86% (HIIT_86_) and 100% (HIIT_100_) of peak speed derived from the Futsal Intermittent Endurance Test (FIET, PS_FIET_) with a total work duration of 16 and 8 min, respectively, implemented over a period of 10 weeks, on the HRV, aerobic fitness, RSA, and neuromuscular performance of young male futsal players. A second aim of this study was to examine the dose-response relationships between accumulated TL and changes in physical and physiological measures. Based on previous studies (Sanchez-Sanchez et al., 2018; Teixeira et al., 2018, 2019) our hypothesis was that the HIIT model with more COD and longer set duration (HIIT_86_) would induce superior improvements on the selected physiological and physical measures than the model with less COD and shorter set duration performed at a higher intensity (HIIT_100_).

## Materials and Methods

### Subjects

The inclusion criteria for the study were regular participation in, at least, 75% of the training sessions during the period of investigation, not suffering from injuries during the same period, and not taking any medication that could alter the outcome of this study. Eleven young male futsal players (mean ± standard deviation; age: 18.5 ± 1.1 years; body mass: 70.5 ± 5.7 kg; height: 1.78 ± 0.07 m) from the U-20 professional futsal team of the first division of Paraná state – Brazil took part in this study. None of the players suffered any injury during the study period and all of them attended more than 75% of the training sessions during the 10 weeks of training. All players and their guardians were informed about the procedures of the study and signed an informed consent form. This study was approved by the local research ethics committee (n° 93777318.0.0000.0121) in accordance with current national and international laws and regulations governing the use of human subjects (Declaration of Helsinki II).

### Experimental Design

**FIGURE 1:**
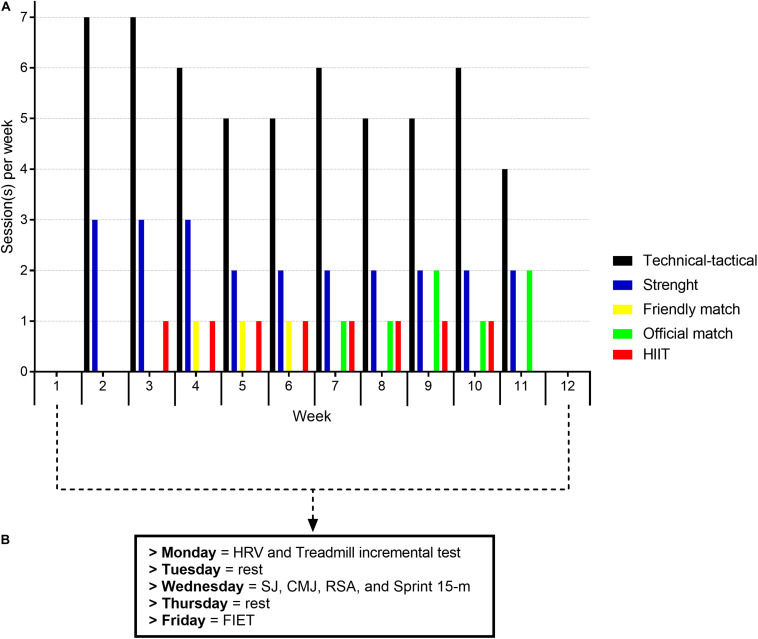
Experimental design with the number of each training/match session type in each week **(A)**, and the testing sessions at pre and post 10 weeks of training **(B)**. HIIT, high intensity interval training; HRV, heart rate variability; SJ, squat jump; CMJ, counter movement jump; RSA, repeated sprint ability test; FIET, Futsal Intermittent Endurance Test.

A parallel 2-group longitudinal experimental study design was performed during 10 weeks from February to April of 2019 (5 weeks of pre-season and 5 weeks during the early in-season). During the study period, players were monitored over 105 training sessions, which were distributed into 16 sessions devoted to HIIT models (8 sessions for each group: HIIT_100_ and HIIT_86_) experimentally implemented for the purposes of this study, 23 sessions to develop strength-power characteristics, 56 sessions dedicated to futsal-specific technical-tactical skills, and 10 matches (3 friendly and 7 official matches; Figure 1A). Before and after the 10-week period, the following physical fitness tests were conducted on three separate days: first day (Monday): resting HRV and incremental treadmill test; second day (Wednesday): vertical jump (CMJ and SJ), straight 15-m sprint, and repeated sprint ability (RSA with COD: 8 x 10+20+10 m) tests; third day (Friday): FIET protocol (Figure 1B). Testing sessions were carried out in a laboratory and on an indoor futsal court, separated by 48 h between each session (Figure 1B), following the procedures of a previous study (Buchheit et al., 2008). Participants were allowed to drink water *ad libitum* during the field testing and training sessions. The internal training load of each player was monitored daily using the session rating of perceived exertion (s-RPE) during all training sessions and matches (Foster et al., 2001; Fanchini et al., 2016).

### Training Intervention

During the intervention period, two different shuttle-run HIIT models were applied based on the individual PS_FIET_ of each player, and both training models were performed once a week. Due to the team’s training schedule, the HIIT sessions started only in the 2nd week and ended in the 9th week of training (last week before the 10th congested week: 2 matches within a 7-day period). All HIIT sessions were carried out before the tactical-technical sessions in the morning period. The HIIT_86_ model consisted of 4 sets of 4-min bouts performed at 86% of PS_FIET_ with 3 min of passive recovery between the sets, whereas the HIIT_100%_ model was composed of 8 sets of 60 s bouts at 100% of PS_FIET_ with 45 s of passive recovery between the sets. Each bout was characterized by 15 s of running effort followed by 15 s of passive rest. Thus, players performed 8 and 2 repetitions of 15 s shuttle runs (with a COD every 3.75 s) during each set of the HIIT_86%_ and HIIT_100%_ models, respectively (Teixeira et al., 2018, 2019). Consequently, all players performed a total of 128 and 64 accelerations and 96 and 48 decelerations/COD actions per training session during the HIIT_86%_ and HIIT_100%_ models, respectively. The average running pace performed by the athletes between the start and return lines for each training model was dictated by a prerecorded audio cue, emitting beeps every 3.75 s (Speaker, Satellite, Taiwan). The distance covered by each athlete during the training sessions was individualized according to their respective PS_FIET_.

### Treadmill Incremental Test

A progressive incremental exercise test was performed on a motorized treadmill (Imbramed ATL, Porto Alegre, Brazil). During the test the treadmill inclination was set at a 1% gradient with an initial speed of 9.0 km/h and then the treadmill speed was increased by 1.0 km/h every minute until volitional exhaustion (Kuipers et al., 2003). The peak speed (PS_TREADMILL_) was calculated according to procedures described elsewhere (Kuipers et al., 2003). Each participant was verbally encouraged to deliver maximum effort during the incremental test. Respiratory gasses were measured breath by breath during the test using a calibrated online metabolic system (K5; COSMED, Rome, Italy). For peak oxygen uptake (VO_2_peak), and first and second ventilatory threshold (VT_1_ and VT_2_, respectively) determination, all gas exchange data were filtered using K5 software (Omnia; COSMED, Rome, Italy) to discard outlier points. Subsequently, the data were reduced to means of 15 s for further analysis. The highest 15 s value of oxygen uptake (VO_2_) was considered as VO_2_peak. For VT_1_ and VT_2_ determination the ventilation/oxygen uptake (VE/VO_2_) and ventilation/carbon dioxide production (VE/VCO_2_) equivalents were used. The first abrupt increase in VE/VO2 without a concomitant increase in VE/VCO2 was considered the VT_1_ (Caiozzo et al., 1982), and the first abrupt increase in VE/VCO2 was considered the VT_2_ (McLellan, 1985). The speed at each threshold was determined.

### Futsal Intermittent Endurance Test (FIET)

The FIET consisted of shuttle-run bouts of 45 m (i.e., 3 × 15 m) performed at progressive speeds until voluntary exhaustion (Castagna and Barbero Álvarez, 2010). Every 45 m, players were allowed to actively rest for 10 s. After each 8 × 45 m bout, players passively rested for 30 s. The starting velocity was set at 9.0 km/h with speed increments of 0.33 km/h during the first 9 × 45 m bouts. After 9 × 45 m bouts, the increment changed to 0.20 km/h every 45 m until exhaustion. The speed was controlled by prerecorded audio cues (Speaker, Satellite, Taiwan). The test was finished when participants did not reach the front line in time with the beeps for 2 consecutive repetitions. The peak speed (i.e., PS_FIET_) reached at the end of the test by the athletes was reported as the performance criterion for the FIET.

### Vertical Jumping Tests

Vertical jump height (cm) was determined using the counter movement jump (CMJ) and the squat jump (SJ). In the CMJ, the participants were instructed to execute a downward movement followed by a complete extension of the legs and were free to determine the countermovement amplitude to avoid changes in jumping coordination. In the SJ, the participants were required to remain in a static position with a 90° knee flexion angle for 3 s before jumping, without any preparatory movements. The CMJ and SJ were executed with the hands fixed on the hips. All jumps were performed on a contact platform (CEFISE, Brazil). A total of 3 attempts were allowed for each jump with a 45 s rest interval between attempts. The best CMJ and SJ attempts were used for further analysis (Bosco et al., 1983).

### Straight 15-m Sprint Test

All players performed three maximal 15 m sprints with at least 2 min of passive rest between the three trials (Cronin and Hansen, 2005). Sprint time was recorded using a photocell system (Microgate, Italy) with timing gates placed at the 0 m (i.e., starting gate) and 15 m marks (i.e., finishing gates). All sprinting tests were conducted on an indoor futsal court, thus eliminating any potential effect of the environmental conditions. The best sprint time was retained for further analysis.

### Repeated Sprint Ability Test

The 40-m RSA test consisted of 8 × 40 m sprints separated by 20 s of passive recovery (Baker et al., 1993). The athlete stared 0.5 m behind the start line and times were recorded electronically via photocells (Microgate, Italy). Before the RSA test, players performed a standardized 5-min warm-up of progressive runs and accelerations that were administered by the team’s physical trainer. Before starting, athletes were instructed to run as fast as possible between two lines placed 20 m apart, with the start/finish line (and the photocells) placed at the midpoint of the course. Each participant sprinted 10 m from the start/finish line to the end of the course, turned 180°, sprinted 20 m to the other end of the course, turned 180°, and sprinted 10 m back through the start/finish line. Following each sprint, the athlete decelerated and walked to the starting line in readiness for the subsequent sprint. Five seconds prior to the next sprint, the athletes assumed the starting position and a 3 s countdown was provided to commence again. The best (RSA_BEST_) and mean sprint times (RSA_MEAN_) were recorded as the performance indices.

### Heart Rate Variability (HRV)

The resting HRV was obtained by time elapsed between two successive R-waves of the QRS signal of the heart rate (R-R intervals) using an RS800cx (Polar Electro, Finland) heart rate monitor. The resting HRV was recorded on Monday mornings at 7:00 a.m., before and after the 10-week period (Figure 1B). During the RR recordings, all players remained at rest for 5 min in the supine position following the standards set by the Task Force (Task Force, 1996). The first 2 min were excluded (signal stabilization), and the remaining 3 min were used to calculate the resting HRV indices. Correction of ectopic beats and/or erroneous signals was performed automatically using the manufacturer’s software (Kubios HRV Analysis, Finland) with a degree of correction < 3% for all recordings, and when necessary, manual correction of artifacts was performed. The resulting R-R intervals were examined in only 1 time-domain index (i.e., root mean square difference of successive normal R-R intervals [rMSSD]). The rMSSD has been reported to reflect vagal modulation and to be related to training-induced effects (Buchheit et al., 2009). The delta change (post – pre training values) in rMSSD (ΔrMSSD) was used for analysis.

### Training Load (TL)

The internal TL was measured using the s-RPE method (Foster et al., 2001). Thirty minutes after the completion of each training session and matches, players were requested to report RPE for the intensity of the training sessions and matches using a 0-100 point RPE scale proposed by Borg and Borg (2002) and recently validated by Fanchini et al. (2016). The 0-100 scale value reported by the players was divided by 10 (i.e., RPE: 75 ÷ 10 = 7.5), and this value was multiplied by session duration, in minutes, to calculate the TL of each training session and matches. When two training sessions were performed on the same day, the TL was summed to create the daily TL. During each training week, the daily TL was summed to create the total weekly TL.

### Statistical Analysis

The analysis was performed using established Bayesian inference methods. The physiological and performance data were analyzed as percentage deltas of pre-measure (Δ% = ((*Post*−*Pre*)/*Pre*)×100) (except for HRV that was analyzed in raw units). Analysis was performed using the linear modeling procedure, with the training models (i.e., HIIT_100_ and HIIT_86_), and the baseline measures centered to the mean of all study subjects included as fixed effects. Additionally, a dose-response analysis was used to verify the relationship of responses to training with the TL and HRV measures. Thus, the training models, delta in the HRV or training load measure, and interaction between training models × HRV/training load were inserted in the model as fixed effects. The Bayesian R^2^ was calculated as an estimate of the proportion of variance explained for new data (Gelman et al., 2019). Model fitting is performed using Markov Chain Monte Carlo (MCMC) methods, more specifically the No-U-Turn (NUTS) sampler implemented in Stan. Student t-distribution (df = 3, μ = 0, and σ = 10) priors were set to be non-informative, so that their influence on the estimates was relatively small (Gelman et al., 2008). Unless otherwise stated, all observed data are reported as means ± standard deviations (SD), and the posterior data as means with 90% highest density credible intervals (CIs) for pre to post changes, and medians with 90% equal tailed CIs for dose-response analysis. The CI represents there is a 90% probability that the parameter is contained within a 90% CI (Morey et al., 2016).

Inferences about the effects were made by interpreting the 90% CI in relation to the region of practical equivalence (ROPE). We specified our ROPE as 0.2 × between-subjects SD (Hopkins et al., 2009). Thus, the ROPEs for VO_2_peak, PS_FIET_, PS_TREADMILL_, VT_2_, VT_1_, RSA_BEST_, RSA_MEAN_, sprint 15-m, CMJ, SJ, and HRV are ± 1.8%, ± 0.9%, ± 1.0%, ± 2.0%, ± 2.3%, ± 0.5%, ± 0.7%, ± 0.9%, ± 1.7%, ± 2.1%, and ± 6.1 ms, respectively. Therefore, an effect was deemed “trivial” when the two bounds of the 90% CI were within the ROPE. Conversely, when the CI overlapped the ROPE the effects were interpreted as “undecided” (Kruschke, 2018). When the two 90% CI bounds were out of the ROPE the effect was deemed as “beneficial” or “harmful”, when positive and negative, respectively; except for RSA_MEAN_, RSA_BEST_, and Sprint 15-m where negative and positive effects were “beneficial” and “harmful”, respectively. Additionally, based on the posterior distributions, we calculated the probability (%) of the effect to be harmful/trivial/beneficial. Statistical analyses were performed using statistical software R (v4.0; R Core Team (2020), Vienna, Austria) and its graphical interface RStudio (v1.2.5). The package “brms” (Bürkner, 2017) allowing fitting of Bayesian multilevel models using “Stan” (Gelman et al., 2015) was used for analysis.

## Results

Descriptive statistics of observed data (mean ± standard deviation [range]) for aerobic, RSA, sprint, and vertical jump performances before (pre-) and after (post-training) the training period (10 weeks) in each HIIT model are presented in Table 1.

**TABLE 1:** Observed means ± SD (minimum – maximum) of physiological, and performance parameters of futsal players in each HIIT model pre and post ten weeks of training and the changes.

**Parameter**	**HIIT_100_ (*n* = 5)**			**HIIT_86_ (*n* = 6)**		
	**Pre**	**Post**	**Δ Pre-Post**	**Pre**	**Post**	**Δ Pre-Post**
VO_2_peak (mL/kg/min)	56.5 ± 5.2 (50.7–62.2)	59.9 ± 4.1 (54.9–64.4)	3.5 ± 1.4 (1.6–4.6)	63.4 ± 3.5 (57.3–66.7)	66.7 ± 2.6 (63.2–70.0)	3.3 ± 1.4 (2.0–5.9)
PS_FIET_ (km/h)	15.7 ± 0.5 (14.8–16.2)	16.4 ± 0.8 (15.4–17.2)	0.8 ± 0.6 (0.2–1.6)	16.5 ± 0.7 (15.6–17.4)	17.0 ± 0.6 (16.0–17.8)	0.5 ± 0.7 (0.00–1.8)
PS_TREADMILL_ (km/h)	16.8 ± 1.4 (15.2–18.0)	17.8 ± 1.0 (16.6–19.1)	1.0 ± 0.7 (0.1–1.8)	17.3 ± 1.5 (15.6–19.1)	18.9 ± 0.3 (18.5–19.3)	1.6 ± 1.2 (0.0–3.1)
VT_2_ (km/h)	14.8 ± 1.6 (13.0–16.0)	15.0 ± 1.7 (13.0–16.0)	0.2 ± 0.8 (−1.0–1.0)	15.4 ± 1.7 (14.0–18.0)	16.8 ± 0.8 (13.0–16.0)	1.3 ± 0.8 (0.0–2.0)
VT_1_ (km/h)	11.5 ± 1.1 (10.0–13.0)	11.7 ± 1.6 (10.0–13.0)	0.2 ± 1.3 (−2.0–1.0)	11.8 ± 1.7 (11.0–15.0)	13.3 ± 1.1 (12.0–15.0)	1.5 ± 1.0 (0.0–3.0)
RSA_BEST_ (s)	8.12 ± 0.20 (7.98–8.47)	8.13 ± 0.22 (7.94–8.50)	0.01 ± 0.07 (−0.11–0.05)	8.28 ± 0.24 (7.86–8.56)	8.06 ± 0.37 (7.47–8.56)	−0.22 ± 0.14 (−0.39–0.00)
RSA_MEAN_ (s)	8.69 ± 0.36 (8.33–9.24)	8.43 ± 0.31 (8.13–8.87)	−0.26 ± 0.10 (−0.37–−0.12)	8.50 ± 0.18 (8.20–8.71)	8.25 ± 0.31 (7.81–8.64)	−0.26 ± 0.20 (−0.44–0.09)
Sprint 15-m (s)	2.50 ± 0.13 (2.35–2.69)	2.42 ± 0.06 (2.32–2.47)	−0.08 ± 0.11 (−0.27–0.00)	2.43 ± 0.08 (2.33–2.53)	2.37 ± 0.09 (2.29–2.54)	−0.05 ± 0.08 (−0.17–0.05)
CMJ (cm)	32.7 ± 1.5 (31.3–34.3)	35.5 ± 1.5 (34.2–37.8)	2.8 ± 1.9 (0.0–4.9)	33.4 ± 3.7 (27.1–37.1)	38.0 ± 4.7 (28.6–41.0)	4.6 ± 2.5 (1.5–8.6)
SJ (cm)	31.2 ± 2.5 (27.9–33.8)	32.4 ± 1.8 (30.5–34.6)	1.2 ± 2.2 (−2.7–2.7)	31.6 ± 4.2 (25.6–36.3)	36.6 ± 4.1 (29.3–40.1)	5.0 ± 4.3 (−1.2–11.7)
HRV (ms)	61.0 ± 21.4 (34.4–80.6)	104.4 ± 20.8 (87.1–137.7)	43.4 ± 40.1 (4.4–103.3)	70.5 ± 38.2 (17.2–120.3)	109.2 ± 32.9 (54.6–150.0)	38.7 ± 25.4 (11.2–86.1)

*All data are means ± SD (minimum – maximum) in raw units. VO_2_peak, peak oxygen uptake; PS_FIET_, peak speed of FIET test; PS_TREADMILL_, peak speed of treadmill incremental test; VT_2_, second ventilatory threshold; VT_1_, first ventilatory threshold; RSA_BEST_, best sprint time in the RSA test; RSA_MEAN_, mean sprint time in the RSA test; Sprint 15-m, best time in 15 meters; CMJ, counter movement jump; SJ, squat jump; HRV, heart rate variability; Δ, change pre to post.*

**FIGURE 2:**
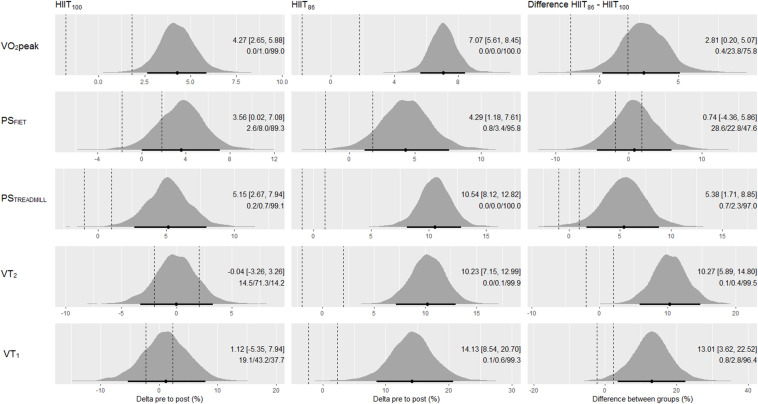
Posterior density distributions and the respective means with 90% credible intervals of the pre to post changes in VO_2_peak, PS_FIET_, PS_TREADMILL_, VT_2_, and VT_1_ (upper to lower panels, respectively) in each HIIT model, and the comparison of changes between models. The effects are adjusted to baseline mean of all study subjects. Black points and error bars are the posterior mean change and 90% credible intervals, respectively. Vertical dashed lines are the lower and upper boundaries of the ROPE (i.e., 0.2× between subjects SD). The texts in each graph are the means (90% CI) and probabilities against ROPE.

Changes for the parameters determined during the FIET and treadmill incremental tests are displayed in Figure 2. The HIIT_100_ model showed clear beneficial effects (i.e., the full 90% CI boundaries out of ROPE) for VO_2_peak and PS_TREADMILL_ measurements. For the HIIT_86_ model, clear beneficial changes occurred in the VO_2_peak, PS_TREADMILL_, VT_2_, and VT_1_. For the HIIT_100_ model, VT_2_ and VT_1_ changes were well supported within the ROPE, nevertheless, these effects are inconclusive because they overlapped the lower and upper ROPE boundaries. For PS_FIET_, both HIIT models presented no clear improvements, although the probabilities were high for improvement (> 89%), low for trivial (< 8%), and negligible for impairment (< 2.6%). Between HIIT models comparisons showed more favorable probabilities in favor of HIIT_86_ than HIIT_100_ in all parameters; however, clearly more favorable changes for the HIIT_86_ model compared to the HIIT_100_ model were observed only for PS_TREADMILL_, VT_2_, and VT_1_ (probabilities > 96%).

**FIGURE 3:**
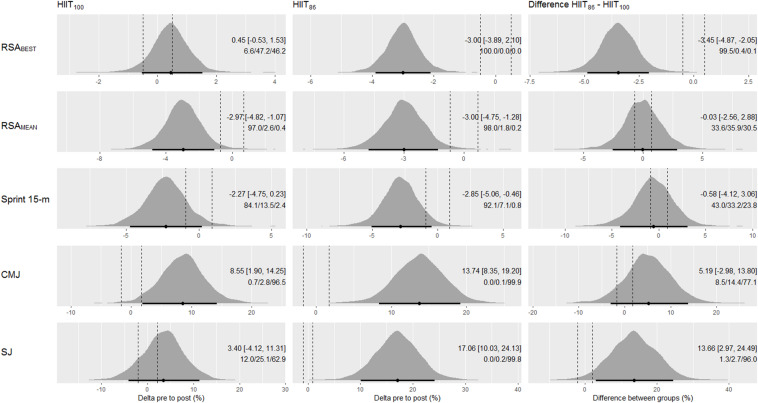
Posterior density distributions and the respective means with 90% credible intervals of the pre to post changes in RSA_BEST_, RSA_MEAN_, Sprint 15-m, CMJ, and SJ (upper to lower panels, respectively) in each HIIT model, and the comparison of changes between models. The effects are adjusted to baseline mean of all study subjects. Black points and error bars are the posterior mean change and 90% credible intervals, respectively. Vertical dashed lines are the lower and upper boundaries of the ROPE (i.e., 0.2× between subjects SD). The texts in each graph are the means (90% CI) and probabilities against ROPE.

Pre to post changes for RSA_MEAN_, RSA_BEST_, 15-m sprint, CMJ, and SJ measures are summarized in Figure 3. The HIIT_100_ model showed clear beneficial effects for RSA_MEAN_ and CMJ measurements, considerable probability of improvement for 15-m sprint time (84%), and inconclusive effects for RSA_BEST_ and SJ. The HIIT_86_ model showed clear beneficial effects for all anaerobic running measurements, except the 15-m sprint time, where a high probability of improvement (92.1%), small/moderate of being trivial (7.1%), and negligible of being harmful (0.8%) were observed. Between HIIT models comparisons showed clearly more favorable changes for the HIIT_86_ than HIIT_100_ model in the RSA_BEST_ and SJ measures (probabilities > 96%). All other effects between model were deemed inconclusive.

**FIGURE 4:**
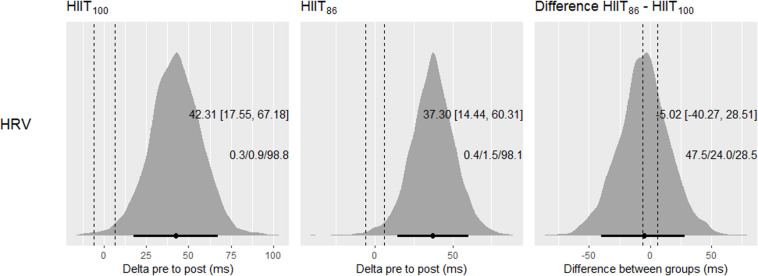
Posterior density distributions and the respective means with 90% credible intervals of the pre to post changes in HRV in each HIIT model, and the comparison of changes between models. Black points and error bars are the posterior mean change and 90% credible intervals, respectively. Vertical dashed lines are the lower and upper boundaries of the ROPE (i.e., 0.2× between subjects SD). The texts in each graph are the means (90% CI) and probabilities against ROPE.

Pre to post change in the HRV revealed clear beneficial changes in both training models, with probabilities > 98.1% (Figure 4). However, no evidence of superiority of one HIIT model over the other was observed.

**FIGURE 5:**
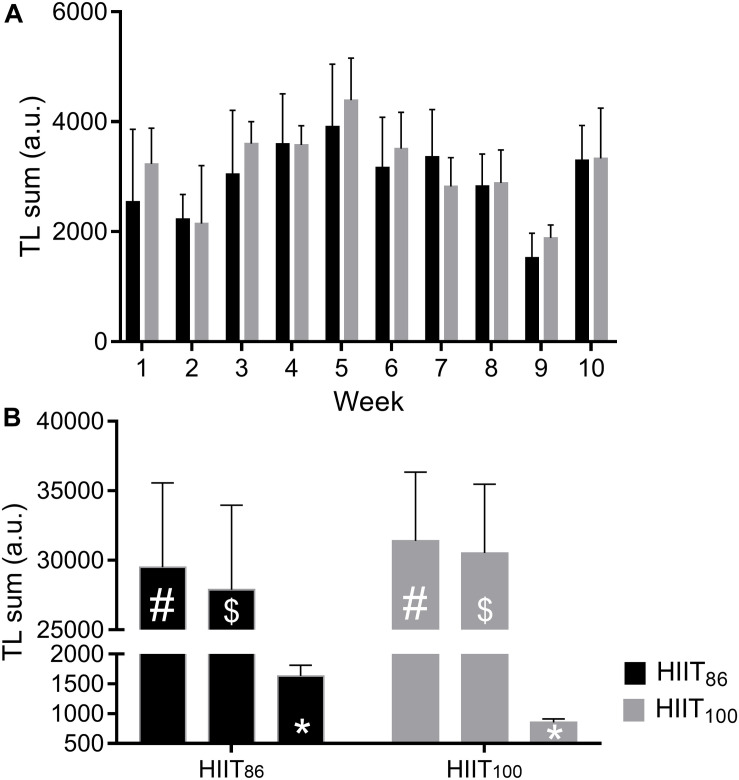
Observed means ± SD of training load sum in each week **(A)** and the accumulated sum over 10 weeks of all training/match sessions [#], all training/match sessions without HIIT [$], and only HIIT sessions [*] **(B)**. Bars and error bars are the mean and SD, respectively. a.u., arbitrary units.

The total weekly TLs for both HIIT_86_ (black bars) and HIIT_100_ (gray bars) models during each training week are presented in Figure 5A. The total accumulated TLs derived from (i) all training sessions and matches (#), (ii) all training sessions and matches without HIIT sessions ($), and (iii) HIIT sessions (^∗^) are presented in Figure 5B. For the entire training period, the total accumulated TL from all training sessions and matches showed a mean difference [90% CI] of −1836 [−8279, 4340] arbitrary units (a.u.) between HIIT_86_ and HIIT_100_ models, with a probability of 55.9% of the TL being lesser in the HIIT_86_ than HIIT_100_. The total accumulated TL of all training sessions and matches without HIIT was lesser in the HIIT_86_ than HIIT_100_ (−2671 [90% CI; −4137, 9004] a.u.), with a probability of TL being lesser in the HIIT_86_ of 76.5%. Contrarily, as expected, the total accumulated TL over the 8 HIIT sessions was two-fold higher in the HIIT_86_ than the HIIT_100_ model (difference: 778 [90% CI; 609, 941] a.u.), with 100% probability of being higher in the HIIT_86_ model.

**FIGURE 6:**
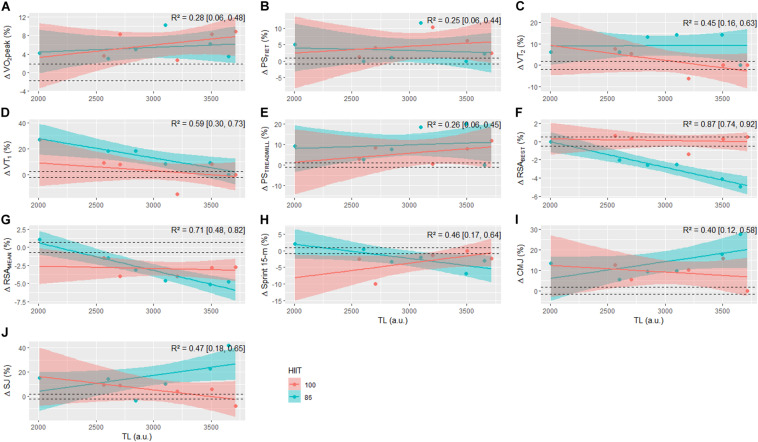
Posterior regression medians with 90% credible intervals between pre-to-post changes in VO_2_peak **(A)**, PS_FIET_ **(B)**, VT_2_ **(C)**, VT_1_ **(D)**, PS_TREADMILL_ **(E)**, RSA_BEST_ **(F)**, RSA_MEAN_ **(G)**, Sprint 15-m **(H)**, CMJ **(I)**, and SJ **(J)** for each HIIT model with training load. R^2^, Bayesian variance explained. Horizontal dashed lines are the lower and upper boundaries of the ROPE (i.e., 0.2× between subjects SD).

**FIGURE 7:**
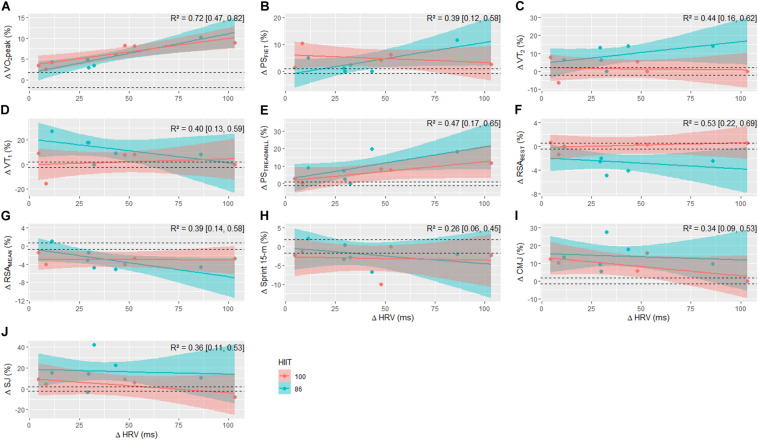
Posterior regression medians with 90% credible intervals between pre-to-post changes in VO_2_peak **(A)**, PS_FIET_ **(B)**, VT_2_ **(C)**, VT_1_ **(D)**, PS_TREADMILL_ **(E)**, RSA_BEST_ **(F)**, RSA_MEAN_ **(G)**, Sprint 15-m **(H)**, CMJ **(I)**, and SJ **(J)** for each HIIT model with the pre-to-post change in HRV. R^2^, Bayesian variance explained. Horizontal dashed lines are the lower and upper boundaries of the ROPE (i.e., 0.2× between subjects SD).

Since the players followed the same training routine, with the exception of the HIIT sessions, the dose-response relationship between RPE-based TL and Δ rMSSD with changes in aerobic, RSA, 15-m sprint, and jump performances was carried out, adding HIIT models as a covariate in the final model. The regression outputs between total weekly TL (10-week average) and ΔrMSSD in addition to HIIT type with changes in performance measures are displayed in Figures 6, 7, respectively. Total weekly TL and HIIT model accounted for 25 to 87% of the variance (i.e., Bayesian *R*^2^) in aerobic fitness, RSA, and power-speed-related performance changes. The explained variance derived from regression models using ΔrMSSD and HIIT type as covariates ranged from 26 to 72%.

## Discussion

The current study aimed to compare the effects of two shuttle run-based HIIT models of varying intensity and total work duration (HITT_86_: 16 min vs. HITT_100_: 8 min) on aerobic, HRV, RSA, and neuromuscular performance outcomes in junior male futsal players. The dose-response relationship between RPE-based TL and changes in performance was also examined. The main findings of this study showed that after 10-weeks of futsal training: (i) the HIIT_86_ model was clearly more effective at improving the PS_TREADMILL_ (Δ = 5.4%), VT_2_ (Δ = 10.0%), VT_1_ (Δ = 13.0%), RSA_BEST_ time (Δ = −3.5%), and SJ height (Δ = 13.7%) than the HIIT_100_; (ii) RPE-based TL in association with HIIT type explained 71% to 87% of the inter-individual variation in RSA performance changes, while the explained variance for the other parameters was smaller (25–59%); and (iii) changes in HRV along with HIIT type accounted for 72% of inter-individual variance in VO_2_peak changes following the training period.

Comparative studies examining the effectiveness of different training models are increasingly needed and recommended to help guide decision-making of strength and conditioning coaches during the planning of training programs with futsal players (Buchheit et al., 2008; Ferrari-Bravo et al., 2008; Sanchez-Sanchez et al., 2018). Training intensity and total work duration are two key variables commonly altered in order to increase the physical capacity of athletes (Buchheit and Laursen, 2013) and, therefore, they need to be well managed during HIIT programming. In the current study, the improvements in physical fitness indices were specific to training type. Our data indicated that the HIIT_86_ model clearly improved (i.e., full 90% CI out of ROPE) the majority of the physical and physiological parameters measured (9 out of 11 parameters; Figures 2, 3, 4) compared to the HIIT_100_ model (5 out of 11 parameters; Figures 2, 3, 4). In addition, the HIIT_86_ model induced larger improvements in aerobic, RSA, and neuromuscular performance outcomes than the HIIT_100_ model. The results presented herein suggest that the HIIT_86_ training, comprising longer sets at a lower intensity, was more effective to enhance performance than the HITT_100_ composed of shorter sets and more intense running efforts. Similarly, Buchheit et al. (2008) also reported greater performance adaptations after HIIT models performed at lower running intensities (i.e., close to 90–95% V_IFT_) in male adolescent handball players. Seiler et al. (2013) also showed that training intensity and total work duration influenced the magnitude of adaptive responses following distinct HIIT models (4 × 4 min; 4 × 8 min; 4 × 16 min) in trained cyclists. On the other hand, current studies published in the literature suggests that HIIT models of different training intensities induced similar performance enhancements in male adults (McGinley and Bishop, 2016; Viaño-Santasmarinas et al., 2018). Some differences between these studies should be addressed. For instance, the total work duration in our training design varied between training groups, while prior studies used an *isotime* approach (i.e., matched-work) only varying exercise intensity (Buchheit et al., 2008; Seiler et al., 2013; McGinley and Bishop, 2016; Viaño-Santasmarinas et al., 2018). In addition, the reference running speed used for training prescription also differed between the cited studies (Buchheit et al., 2008; Viaño-Santasmarinas et al., 2018). Thus, comparison between the current results and those observed in prior studies should be interpreted with caution due to differences in sample characteristics (e.g., chronological age), methodological issues, sport modality, training demands, and performance levels.

It is well known and accepted that aerobic fitness and RSA performance are two discriminant physical qualities of the competitive level in futsal (Álvarez et al., 2009; Pedro et al., 2013; Ayarra et al., 2018). Thus, training strategies targeting the development of these physical qualities simultaneously are essential. Our findings indicating the superiority of HIIT_86_ over HIIT_100_ at improving aerobic fitness, RSA, and vertical jump performance suggest that this training type (submaximal runs at 86% PS_FIET_ and longer sets) should be preferentially used with young futsal players. The specific adaptations in physical performance following the HIIT_86_ and HITT_100_ models could potentially be related to differences in total work duration between the models (Teixeira et al., 2018, 2019). Due to its higher total running volume (16 vs. 8 min), the HIIT_86_ implies a greater number of COD performed in a single session (96 vs. 48 turns), increasing the total time that athletes spent accelerating per running bout compared to the HIIT_100_ model. Akenhead et al. (2014) showed that the number of turns and time spent accelerating (>± 1 m⋅s^–2^) are linearly related during shuttle run drills. Prior research has indicated that accumulated individual acceleration load is positively associated with changes in aerobic fitness and neuromuscular measures in professional soccer players (Clemente et al., 2019). Given that acute and chronic responses to training are dependent on acceleration load accumulated during multidirectional drills (Akenhead et al., 2014; Clemente et al., 2019), practitioners and coaches can increase or decrease the acceleration load accumulated by athlete during shuttle run-based HIIT varying either set duration as in this study or COD frequency per running bout (Sanchez-Sanchez et al., 2018; Teixeira et al., 2018, 2019). To date, to the best of our knowledge, this is the first study demonstrating that a HIIT model with more directional changes leads to superior gains in physical performance in a sample of aerobically well-trained male futsal players (VO_2_peak > 55 mL/kg/min at baseline). Previous studies published on this research topic did not show any further performance gain after different shuttle run training models varying the number of COD required per running bout in male soccer and basketball players (da Silva et al., 2015; Attene et al., 2016). Of interest, the same HIIT_86_ model used here was also applied in a sample of female futsal players (VO_2_peak: 47–49 mL/kg/min at baseline) (Teixeira et al., 2018, 2019). The current and prior studies (Teixeira et al., 2018, 2019) showed a greater improvement in PS_TREADMILL_ and RSA performance after HIIT_86_ model. This demonstrates the consistency and effectiveness of this training model (HIIT_86_) to improve these physical qualities in age-matched male and female futsal players. At the same time, male and female futsal athletes of similar ages can display distinct neuromuscular performance adaptations (i.e., changes in SJ and CMJ height) following HIIT_86_, with male athletes in the current study being more responsiveness (Δ = 13–17%) than female athletes (Δ = 8–9%) in the study of Teixeira et al. (2018). Therefore, it should be highlighted that any generalization of our findings to other samples of futsal (male or female; young or adult) or other indoor team sports (e.g., basketball and handball) would be a hasty inference, since differences related to age, gender and sport demand in terms of workload and training content distribution during a typical training period could influence the effectiveness of this HIIT_86_ model in these other sports scenarios.

From the present results on RPE-based TL and changes in physical performance, it is possible to make inferences about the dose-response relationship during the training process. The present study suggests that TL in addition to training type (HIIT_86_ and HIIT_100_) accounted for a large portion of the inter-individual variance in RSA (71–87%), 15-m Sprint (46%), and vertical jump (40–47%) performance changes. A positive linear dose-response relationship between TL and changes in these performance measures was found for the HIIT_86_ model, while no (RSA performance) or negative (sprint and vertical jump) relationships were identified for the HIIT_100_ model. These findings are of practical relevance for practitioners and coaches. First, players in both HIIT_86_ and HIIT_100_ models with a similar total TL displayed a distinct adaptive response (especially for RSA_BEST_ and SJ performance), highlighting that the quality/specificity of the training stimuli is the most relevant component of the training process (Sanchez-Sanchez et al., 2018). In this case, the increased number of COD in the HIIT_86_ model (longer sets) may have been decisive to induce superior gains in performance. Second, players who accumulated higher training loads in the HIIT_86_ model demonstrated the largest improvements in RSA, 15-m Sprint, and vertical jump performance, while the opposite was observed for the HIIT_100_ model. Although these results cannot be easily explained from the data analysis employed in our study, it is possible to suggest that the training loads derived from other training strategies (technical-tactical, strength-power, friendly and official matches) may have influenced the players’ adaptive response to training. For instance, players in the HIIT_100_ model tended to accumulate a higher (with a 73% probability) TL derived from these other training contents (i.e., not involving HIIT sessions) than the HIIT_86_ model (Figure 5).

The explained variances derived from regression models (TL and HIIT type inserted as covariates) for the changes in maximal (VO_2_peak, PS_FIET_, PS_TREADMILL_) and submaximal (VT_2_ and VT_1_) aerobic performance measures were considered low (25–28%) and moderate (45–59%), respectively. Contrary to what was observed in the anaerobic and jump performance measures, the dose-response relationship for aerobic performance outcomes did not show a distinct pattern between HIIT models (i.e., regression slopes in contrary directions). Of note, positive and negative/null relationships were observed between accumulated TL and changes in maximal (VO_2_peak and PS_TREADMILL_) and submaximal (VT_2_ and VT_1_) aerobic indices, respectively. Several studies have demonstrated a linear dose-response relationship between RPE-based TL and changes in aerobic performance indicators in team sports athletes (Oliveira et al., 2013; De Freitas et al., 2015; Clemente et al., 2019; Ellis et al., 2020; Nakamura et al., 2020). Interestingly, a recent study conducted by Ellis et al. (2020) also found a negative weak association between TL and changes at fixed lactate thresholds of 2 and 4 mmol/L (Bayesian *r* = −0.17 and −0.16, respectively) after 6 weeks of pre-season in a sample of male junior soccer players. Furthermore, in agreement with our data, a small positive relationship was verified between TL and PS_TREADMILL_ changes (Bayesian *r* = 0.37) in the previously cited study (Ellis et al., 2020). It should be highlighted that the present study included 2 different shuttle-run HIIT types, so we chose to analyze the dose-response data with an interaction between TL and HIIT type. Thus, our Bayesian R^2^ values consider both the relationship of the response to the TL and the HIIT model. Therefore, comparisons with the *R*^2^ from other studies should be performed with caution to avoid misinterpretations, since they only used the TL as a covariate.

An interesting finding from our study to be highlighted was that similar improvements in the resting HRV were noticed after both HIIT models (HIIT_86_ and HIIT_100_) outlined here using PS_FIET_ as the reference speed to calibrate running distance. Although improved resting HRV after a period of futsal training has been previously documented in the literature (Oliveira et al., 2013; De Freitas et al., 2015; Nakamura et al., 2020), our data reinforce the effectiveness of shuttle run HIIT training models to induce positive adaptations in the cardiac autonomic function of young futsal players. This assumption was previously confirmed by Buchheit et al. (2008) who showed that HIIT models are preferable compared to repeated all-out sprint training methods, since its effects on cardiac autonomic function were significantly more pronounced in young handball players. The resting HRV, a non-invasive assessment of cardiac autonomic modulation, has also been constantly related to aerobic fitness indices and high-intensity running performance changes in team sport athletes (De Freitas et al., 2015; Esco et al., 2016; Nakamura et al., 2020). The present study showed a positive relationship between changes in resting HRV (i.e., rMSSD) and VO_2_peak after the training period, suggesting that players in both HIIT models with greater increases in resting rMSSD demonstrated the largest increments in VO_2_peak. Of interest, changes in resting HRV and HIIT types accounted for 72% of the inter-individual variance in VO_2_peak changes in our sample. Another two studies performed with futsal players also showed that an enhanced vagal modulation (inferred by an increase in resting rMSSD value) was largely positively correlated (*r* = 0.62 and 0.64) with improvements in Yo-Yo IR1 performance (De Freitas et al., 2015; Nakamura et al., 2020). These explained variances (38–40%) are, at least in part, like those observed in our study for the ΔPS_FIET_ (39%) and ΔPS_TREADMILL_ (47%) (Figure 7). Collectively, the current and previous results indicate that resting rMSSD may be a simple and sensitive indicator to monitor changes in physical fitness during training.

One of the strengths of this study was to show how different HIIT models associated with other training components can influence the adaptive responses of players from the same futsal team. In this scenario, where few traditional HIIT sessions are planned by the team technical staff due to the matches schedule and the importance given to technical-tactical and strength/injury prevention sessions, the selection of training stimuli in HIIT sessions is key to maximizing subsequent performance adaptations. From a practical perspective, strength and conditioning specialists should consider spending more time in less intense shuttle run HIIT sessions with more COD than in more intense and shorter sessions with fewer directional changes.

The main limitation of the present study was the small sample size (*n* = 11). This can be justified by the low number of players who are part of a futsal squad (12–15 players). It would be extremely difficult to monitor more than one futsal team at one time. However, Bayesian analysis is better suited for making inferences on small sample sizes, as the MCMC methods used to produce posterior distributions do not depend on asymptotic behavior in the same way that traditional frequentist methods do (Kadane, 2015). Additionally, Bayesian inferences are more intuitive by posterior probability distributions of parameters (Kruschke, 2018). Nevertheless, a challenge in Bayesian statistics is the necessity to impose a prior knowledge in the parameters (i.e., prior distributions). In this way, we used non-informative default priors of the brms package (Bürkner, 2017). Therefore, the priors had little influence on the results (Gelman et al., 2008). Another point related to the analysis is that in team sports, such as futsal, there is no ROPE directly linked to performance. Thus, we used a ROPE of 20% between subjects SD [i.e., Cohen’s *d* standardized mean differences transformed to percent units (Hopkins et al., 2009; Kruschke, 2018)] to infer about the substantiality of our results. Also, we acknowledge that the use of a single day HRV records at pre and post-training period is in disagreement with recent statements suggesting a minimum of 3 randomly selected HRV measurements per week (Plews et al., 2014; Nakamura et al., 2020). Another limitation of the present study is that no objective external load measure was used. A recent prior study suggested that decelerations, number of sprints, and distance covered should be considered to better discriminate the physical load of elite futsal players (Ribeiro et al., 2020). Future studies should consider using larger samples and adding more objective measures to run multiple linear regressions to explain the variations in determinant fitness variables.

## Conclusion

This study showed that those players who underwent 8 shuttle run HIIT sessions at 86% PS_FIET_ had superior gains in aerobic, RSA, and neuromuscular performance measures than those who trained at 100% PS_FIET_ during a typical 10-week training period. In addition, the variance explained by the TL along with the HIIT type was clearly larger for the changes in RSA performance outcomes than that observed for aerobic and neuromuscular performance changes. Finally, monitoring resting HRV could be a suitable tool to track changes in VO_2_peak, since temporal alterations in HRV are strongly related to VO_2_peak changes.

## Data Availability Statement

The raw data supporting the conclusions of this article will be made available by the authors, without undue reservation.

## Ethics Statement

The studies involving human participants were reviewed and approved by this study was approved by the local research ethics committee (n° 93777318.0.0000.0121). Written informed consent to participate in this study was provided by the participants’ legal guardian/next of kin.

## Author Contributions

FC participated in the design of the study, data collection, data organization, and drafted the manuscript. FB participated in the data organization, and performed the statistical analysis, interpretation, and discussion of results. LF and LB contributed with support of materials and data collection. AT participated in the design of the study and interpretation and discussion of results. RHN contributed to the design of the study and interpretation of results. LG contributed to the design of the study, interpretation and discussion of results, and coordination of project. All authors contributed to the writing and approved the final version.

## Conflict of Interest

The authors declare that the research was conducted in the absence of any commercial or financial relationships that could be construed as a potential conflict of interest.
